# Cytochrome *b*5 reductases: Redox regulators of cell homeostasis

**DOI:** 10.1016/j.jbc.2022.102654

**Published:** 2022-10-29

**Authors:** Robert Hall, Shuai Yuan, Katherine Wood, Mate Katona, Adam C. Straub

**Affiliations:** 1 Heart, Lung, Blood and Vascular Medicine Institute, University of Pittsburgh, Pittsburgh, Pennsylvania, USA; 2 Department of Pharmacology and Chemical Biology, University of Pittsburgh, Pittsburgh, Pennsylvania, USA; 3 Center for Microvascular Research, University of Pittsburgh, Pittsburgh, Pennsylvania, USA

**Keywords:** cytochrome b5 reductase, redox, oxidative stress, nitric oxide, coenzyme Q, AFR, ascorbate free radical, CoQ, coenzyme Q, CYB5R, cytochrome-b5 reductase, FAD, flavin adenine nucleotide, mARC, mitochondrial amidoxime reducing component, Mtln, mitoregulin, NO, nitric oxide, RCM, recessive congenital methemoglobinemia, sGC, soluble guanylate cyclase, VDAC1, voltage-dependent anion-selective channel 1

## Abstract

The cytochrome-*b*_5_ reductase (CYB5R) family of flavoproteins is known to regulate reduction-oxidation (redox) balance in cells. The five enzyme members are highly compartmentalized at the subcellular level and function as “redox switches” enabling the reduction of several substrates, such as heme and coenzyme Q. Critical insight into the physiological and pathophysiological significance of CYB5R enzymes has been gleaned from several human genetic variants that cause congenital disease and a broad spectrum of chronic human diseases. Among the CYB5R genetic variants, CYB5R3 is well-characterized and deficiency in expression and activity is associated with type II methemoglobinemia, cancer, neurodegenerative disorders, diabetes, and cardiovascular disease. Importantly, pharmacological and genetic-based strategies are underway to target CYB5R3 to circumvent disease onset and mitigate severity. Despite our knowledge of CYB5R3 in human health and disease, the other reductases in the CYB5R family have been understudied, providing an opportunity to unravel critical function(s) for these enzymes in physiology and disease. In this review, we aim to provide the broad scientific community an up-to-date overview of the molecular, cellular, physiological, and pathophysiological roles of CYB5R proteins.

The cytochrome-*b*_5_ reductase (CYB5R) family of enzymes, consisting of five members (1, 2, 3, 4, 5), is a group of flavoprotein reductases that catalyze the transfer of electrons from NADH, generally through an electron carrier such as cytochrome b_5_ (CYB5), to the final substrate (1). CYB5R3, which is abundantly and ubiquitously expressed across cell types, has been studied extensively throughout the past half decade (1, 2, 3, 4, 5, 6, 7, 8, 9). Structurally, CYB5R3 has been crystallized, revealing a “clam shell-like structure” with two critical structural domains: an NADH and [a] flavin adenine dinucleotide (FAD)-binding domain (Fig. 1). These two domains are held together by a linker region, which plays an important role in maintaining the two domains in the correct orientation and in close proximity to help facilitate electron transfer (10). CYB5R3 has been shown to reduce several critical substrates, such as heme and coenzyme Q (CoQ) (3, 11, 12). However, the other CYB5R family members have not been comprehensively studied. Therefore, much of our understanding of these proteins is based on our preexisting knowledge of CYB5R3 and the extensive sequence similarity between reductases within the CYB5R family (Fig. 2). The sequences of the five reductases are conserved, particularly within the FAD- and NADH-binding domains (Fig. 2), pointing to the importance of the interplay between these domains in the CYB5R family (13). The flavin-binding domain is necessary for CYB5R stability and function (14, 15). Interestingly, among the five reductases, CYB5R1, CYB5R2, and CYB5R3 are the most structurally alike, sharing nearly indistinguishable motifs in both the FAD- and NADH-binding domains (Fig. 2). By contrast, CYB5R3 and CYB5R5 are the least analogous with a 27.87% sequence identity. Based on their primary sequences and predicted structures using Alpha fold (16), it is evident that CYB5R4 and CYB5R5 are the most structurally unique in the CYB5R family. Notably, CYB5R4 contains its own heme-binding domain (Fig. 2), akin to the cytochrome b_5_ carriers CYB5A and CYB5B, which is purportedly essential for electron transfer to target substrates (17, 18). Despite our extensive knowledge of CYB5R3, numerous questions pertaining to the other CYB5R family members remain unanswered. In this review, we lay out a detailed and compendious synopsis of CYB5R enzyme biology with the goal of highlighting salient contributions of these reductases and opportunities for future studies investigating their roles in cell signaling, physiology, and disease.

**Figure 1:**
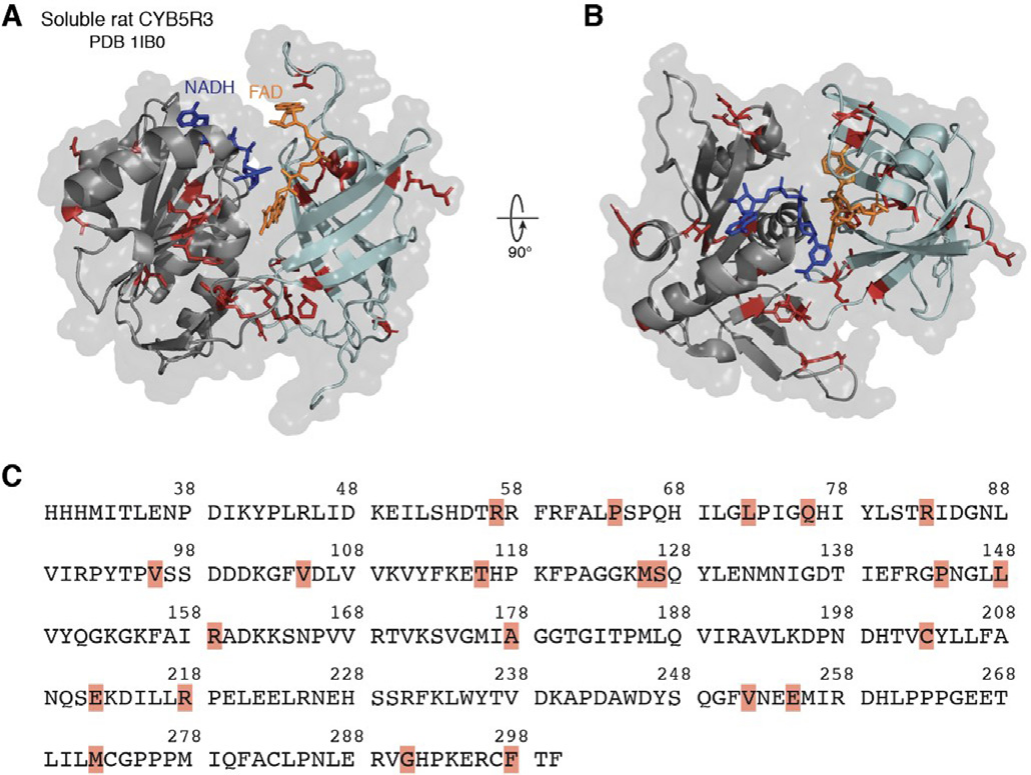
**Crystal structure and amino acid sequence of soluble rat CYB5R3 (PDB entry 1IB0).** The side view (*A*) and top view (*B*) are depicted with FAD (*orange*) and NADH (*blue*) bound. The segments of the protein highlighted *red* illustrate clinically relevant mutations that have already been discovered in the literature. The position of these mutations in the CYB5R3 amino acid sequence is provided below (*C*). The *orange* and *dark blue* molecules depict bound FAD and NADH, respectively. The structure was constructed and visualized in the PyMOL software. FAD, flavin adenine nucleotide.

**Figure 2:**
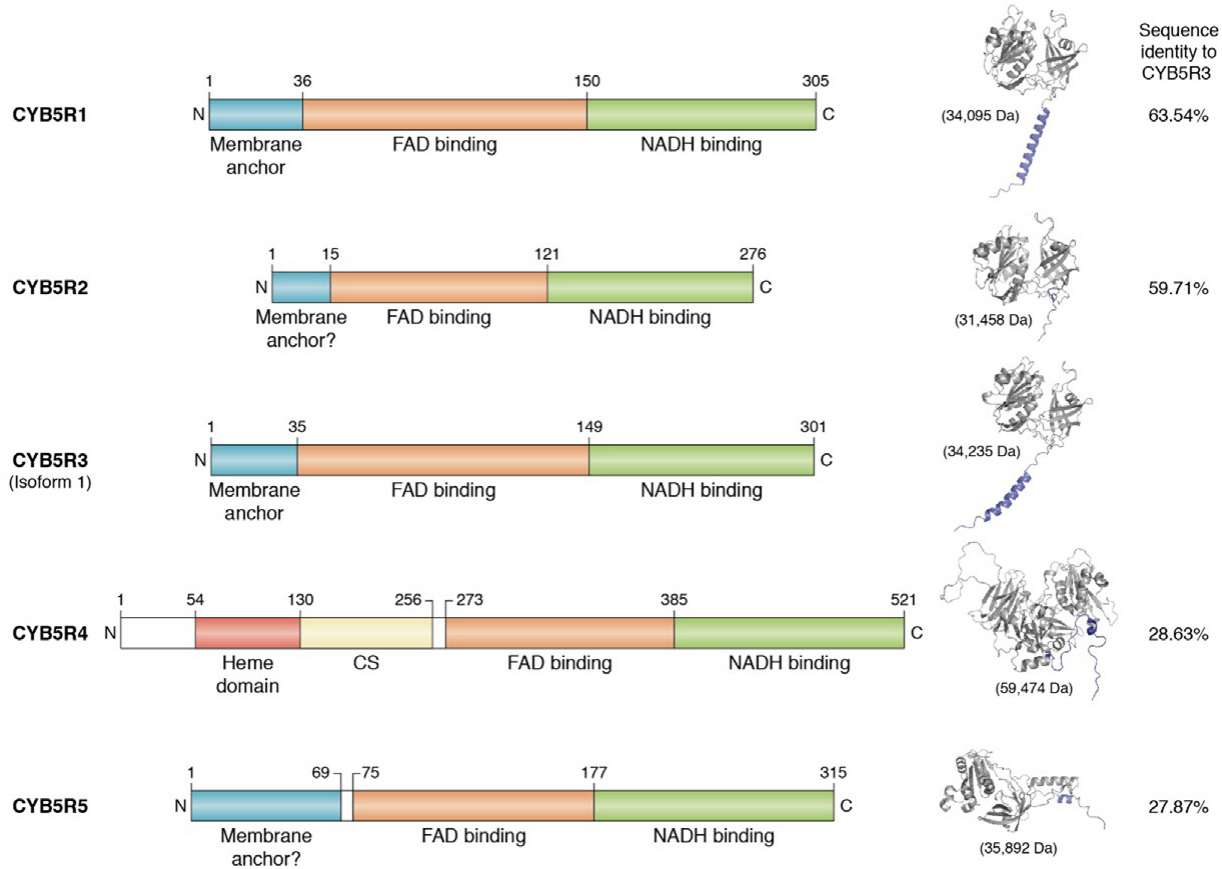
**Linear amino acid sequence (*left*) and crystal structure (*right*) of each CYB5R family member.** Each structure depicts the purported membrane-bound isoform except for CYB5R4, which exists only as a soluble protein. Each structure was obtained from the AlphaFold protein structure database (16). The UniProt IDs are as follows: CYB5R1 – Q9UHQ9 (human); CYB5R2 – Q6BCY4 (human); CYB5R3 – P00387 (human); CYB5R4 – Q7L1T6 (human); CYB5R5 – Q6IPT4 (human). CYB5R, cytochrome-b5 reductase.

## CYB5R1

To date, our knowledge on CYB5R1 function and physiology remains scant. A previous study reported *CYB5R1* mRNA enrichment in human skeletal muscle and predicted protein localization to the mitochondria, plasma membrane, and endoplasmic reticulum (Table 1) (19). CYB5R1 is a 34 kDa protein that harbors a 36-amino acid alpha-helical membrane anchor at the *N*-terminus, permitting CYB5R1 attachment to membranes. CYB5R1 structural analysis discloses conserved structural motifs in both the FAD- and NADH-binding domains, comparable to CYB5R3 (Fig. 2). These conserved structural motifs emerge from similar amino acid sequences, where CYB5R1 and CYB5R3 share a 63.54% sequence identity, the highest in the CYB5R family. CYB5R1 is also structurally homologous to CYB5R2, where there is a 58.82% sequence similarity between the two enzymes. Mass spectrometry analyses have identified numerous posttranslational modifications (PTMs) distinct to CYB5R1, most notably phosphorylation at site Y84 (20). This residue is situated in the FAD-binding domain; thus, one might postulate that phosphorylation at Y84 may optimally govern electron transfer efficiency by the FAD molecule. Furthermore, K167, positioned within the linker region bridging the NADH and FAD domains, has been identified as an acetylation site (21). Located in the linker region, one might speculate that acetylation at K167 could induce a conformational change in both the NADH and FAD domains, thereby impacting CYB5R1 activity. To date, it is uncertain whether crosstalk between CYB5R1 phosphorylation and acetylation occurs. For instance, does phosphorylation at Y84 directly influence the addition or removal of the acetyl group at K167 and vice versa? Understanding the potential crosstalk between posttranslational modifications could shed light on how CYB5R1 activity is “turned on” or “turned off” under disparate pathophysiological conditions. Notably, it is uncertain whether these posttranslational modifications are linked with CYB5R1 activity or specific pathological conditions. Answering these questions could open doors for targeted therapeutic development.

**Table 1:** A comprehensive table indicating the subcellular location, cofactors, known roles within and outside of the cardiovascular (CV) system, and highest tissue expression for each of the five reductases in the CYB5R family

Reductase	Subcellular location	Cofactors	Known role in CV system	Known role outside of CV system	Human tissue(s) with highest mRNA expression	References
NADH-Cytochrome b_5_ reductase 1	Mitochondria, extracellular space, plasma membrane	NADH, FAD	Unknown	Induction of lipid peroxidation and ferroptosis, lipid desaturation	Skeletal muscle	(19, 22, 137)
NADH-Cytochrome b_5_ reductase 2	Nucleus, cytosol	NADH, FAD	Unknown	Protection against prostate, nasopharynx, and colorectal cancer	Testis, tibial nerve	(19, 31, 32, 33, 34, 35)
NADH-Cytochrome b_5_ reductase 3	Mitochondria, plasma membrane, ER, cytosol	NADH, FAD	Heme reduction, lipid regulation, cholesterol biosynthesis, CoQ regulation, sGC regulation, protection against lipid peroxidation	Protection of pancreatic beta cells against oxidative stress, drug metabolism	Artery, aorta, adipocyte	(1, 3, 4, 5, 7, 8, 19, 36, 45, 49, 62)
NADH-Cytochrome b_5_ reductase 4	ER, cytosol	NADPH, NADH, FAD, heme	Unknown	Protection of pancreatic beta cells against oxidative stress, regulation of satiety and feeding behavior, fatty acid desaturation, iron homeostasis	Whole blood cells (peripheral blood mononuclear cells, platelets, monocytes, T-lymphocytes, granulocytes)	(19, 129, 131, 132, 133, 134)
NADH-Cytochrome b_5_ reductase 5	Nucleoplasm, ER	NADH, FAD	Unknown	Mitigation of oxidative stress in colon polyps	Low tissue specificity	(19, 136)

A recent publication demonstrated that CYB5R1, localized to the ER membrane, functions in tandem with NADPH-cytochrome p450 reductase (POR) to catalyze lipid peroxidation and ferroptosis execution in HeLa cells (22). Ferroptosis is an iron-dependent form of cell death triggered by intracellular iron (Fe^2+^) accumulation and elevated hydrogen peroxide (H_2_O_2_) levels, which together lead to lipid peroxidation and execution of cell death (23). Cooperatively, these two enzymes are sufficient to induce lipid peroxidation and ferroptosis by reducing intracellular molecular oxygen, leading to hydrogen peroxide formation and subsequent membrane lipid oxidation *via* the Fenton reaction (22). The Fenton reaction is a reaction between free Fe^2+^ and H_2_O_2_ that yields a hydroxyl radical, a potent oxidant that attacks polyunsaturated fatty acids in membranes, ultimately leading to membrane rupture and cell death. Importantly, the authors noted that ferroptosis was mostly POR-dependent due to this enzymes capacity to generate a greater concentration of H_2_O_2_ (22); thus, it appears CYB5R1 plays a lesser role in ferroptosis execution in HeLa cells. Interestingly, Woischke *et al.* (24) revealed a potential link between CYB5R1 overexpression and the risk of developing colorectal cancer, a seemingly contradictory finding to the ferroptosis study aforementioned. If CYB5R1 induces ferroptosis, one might expect that cancer cells would die; however, Woischke *et al.* assert that CYB5R1 is protective for colorectal cancer cells. Therefore, follow-up studies should be conducted to clear these discrepancies. As a whole, the connection between ferroptosis and cancer has been delineated in the literature (25, 26), where ferroptosis-inducing drugs show promise in reducing tumor size and improving efficacy of chemotherapeutic drugs (27). Given CYB5R1’s reported role in ferroptosis and colorectal cancer, therapeutics targeting CYB5R1 could be beneficial in treating those suffering from certain cancers. The link between CYB5R1 and ferroptosis could also be important in various cell types, especially primary cells, and outside the realm of cancer research. This prospect should be investigated further to better understand the regulation of ferroptosis by CYB5R1.

Beyond cancer, *CYB5R1* transcript levels are significantly upregulated in retina samples collected from patients with diabetic retinopathy, a disease characterized by aberrations in the retinal microvasculature and blindness (28). Importantly, similar changes in RNA were discerned in mice exhibiting diabetic retinopathy. The authors propose that CYB5R1 is localized to the mitochondria, where the enzyme likely plays a pivotal role in oxidative phosphorylation and ROS generation (29). This suggests that CYB5R1 might be involved in mitochondrial oxidative stress pathways that factor into the development of diabetic retinopathy. By investigating a gene co-expression network in a human diabetic retinopathy dataset, the authors further surmise that CYB5R1 is acting on complex 1 of the electron transport chain (29). Since CYB5R1 is an electron donor, it is possible that overexpression of CYB5R1 elicits electron leak during the CYB5R1 electron transfer reactions which results in the reduction of molecular oxygen to generate superoxide. These observations stipulate that models of diabetic retinopathy may be advantageous for understanding mechanisms by which CYB5R1 is involved in triggering oxidative stress in the retinal microcirculation.

Beyond changes in expression levels of CYB5R1, it is unclear whether human genetic coding variants associate with human disease. Scanning of the human genome database revealed that an N44S mutation, positioned at the interface between the FAD domain and the membrane anchor, has a 44% allele frequency in East Asians and only 3% in populations with European ancestry (30). Thus, future studies aimed at crystallizing both WT and CYB5R1 mutants could shed light on the structural and functional consequences of CYB5R1 mutations in human health and disease across ethnicities.

## CYB5R2

In addition to CYB5R1, CYB5R2 has also been understudied. *CYB5R2* mRNA has been predicted to be enriched in the testis and tibial nerve in humans (Table 1) (19). CYB5R2 is a 31 kDa protein speculated to have nucleus and cytosolic confinement. When comparing the amino acid sequence of CYB5R2 to the other CYB5R family members, CYB5R2 has a 59.71% and 58.82% sequence similarity with CYB5R3 and CYB5R1, respectively. By contrast, CYB5R2 shares only a 28.35% and 28.82% sequence similarity with CYB5R4 and CYB5R5, respectively. CYB5R1, CYB5R2, and CYB5R3 all include similar structural motifs (Fig. 2)—namely, an FAD-binding domain consisting of six antiparallel β-sheets and one α-helix, an NADH domain comprised of five β-strands and four α-helices, a linker region consisting of three antiparallel β-sheets, and an alpha-helical membrane anchor (10). Whether CYB5R2 harbors a membrane anchor remains uncertain. CYB5R2 is phosphorylated at four amino acid sites (21). Two of them, S41 and T157, are situated in the FAD and NADH domains, respectively. On the other hand, two phosphorylation sites, T145 and N131, are situated within the linker region connecting the two domains. This suggests that the linker region is tightly regulated by phosphorylation to perpetuate interdomain interactions, stability, and biological activity. Future studies devoted to understanding the structural and functional implications of CYB5R2 modulation through phosphorylation and the crosstalk between different phosphorylation sites could provide invaluable insight into CYB5R2’s role in physiology and disease.

The functional role of CYB5R2 in the cardiovascular system has not been investigated. However, one study conducted by Franceschini *et al.* (31) identified a single nucleotide-polymorphism located 10 kilobases downstream of the *CYB5R2* gene that associated with elevated blood pressure exclusively in those with African ancestry. Yet, whether CYB5R2 plays a role in blood pressure regulation has yet to be determined. Aside from this study, CYB5R2 has almost exclusively been investigated in the context of cancer. It was recently shown that *CYB5R2* may act as a tumor suppressor gene in human nasopharyngeal cancer (32). This study revealed that CYB5R2 expression was reduced and the *CYB5R2* promoter was hypermethylated in nasopharyngeal tumors. *CYB5R2* promoter methylation associated with lymph node metastasis, suggesting that downregulation of CYB5R2 protein expression and methylation of its promoter in nasopharyngeal epithelium could potentially be used to forecast lymph node metastasis. Moreover, reconstitution of CYB5R2 in nasopharyngeal cancer cell lines suppressed cell proliferation and migration (32). Intriguingly, an unrelated study corroborated these findings. CYB5R2 was shown to upregulate genes that negatively impact angiogenesis in nasopharyngeal cancer cells and downregulate expression of vascular endothelial growth factor, thereby suppressing angiogenesis and tumor migration (33). These studies present unique findings that could lead to the development of targeted therapeutics, where enhancing CYB5R2 expression or activity could be a novel therapeutic approach for treating nasopharyngeal cancer.

Jo *et al.* discovered a potential protective role for CYB5R2 in colorectal cancer by acting as a tumor suppressing gene. This group found that two separate colorectal cancer cell lines harbor a somatic frameshift mutation in the *CYB5R2* gene, resulting in a truncated protein (34). However, this investigation did not interrogate the clinical and histopathological parameters associated with colorectal tumors possessing the truncated CYB5R2 protein. These novel findings present a unique role of CYB5R2 in the pathogenesis of colorectal cancer and could lead to potential therapeutic interventions. However, further studies are needed to further illuminate the clinical implications of *CYB5R2* frameshift mutations in colorectal cancer.

Finally, CYB5R2 has been implicated in prostate cancer. Akin to the nasopharyngeal cancer study, *CYB5R2* was found to be hypermethylated in prostate cancer in a tissue-specific manner, thereby facilitating prostate pathogenesis (35). This implies that CYB5R2 may protect against prostate cancer. It would be curious to dissect the mechanisms responsible for the direct or indirect methylation of the *CYB5R2* promoter that enable neutralization of the CYB5R2 gene. This study illustrates that epigenetic dysregulation of critical regulatory components, such as CYB5R2, can favor prostate carcinogenesis. These new findings demonstrate a potential role of CYB5R2 in mitigating the progression of prostate cancer, which could lead to the development of therapeutics that effectively target the expression or stability of CYB5R2.

## CYB5R3

### Structure and electron transfer reaction

**Figure 3:**
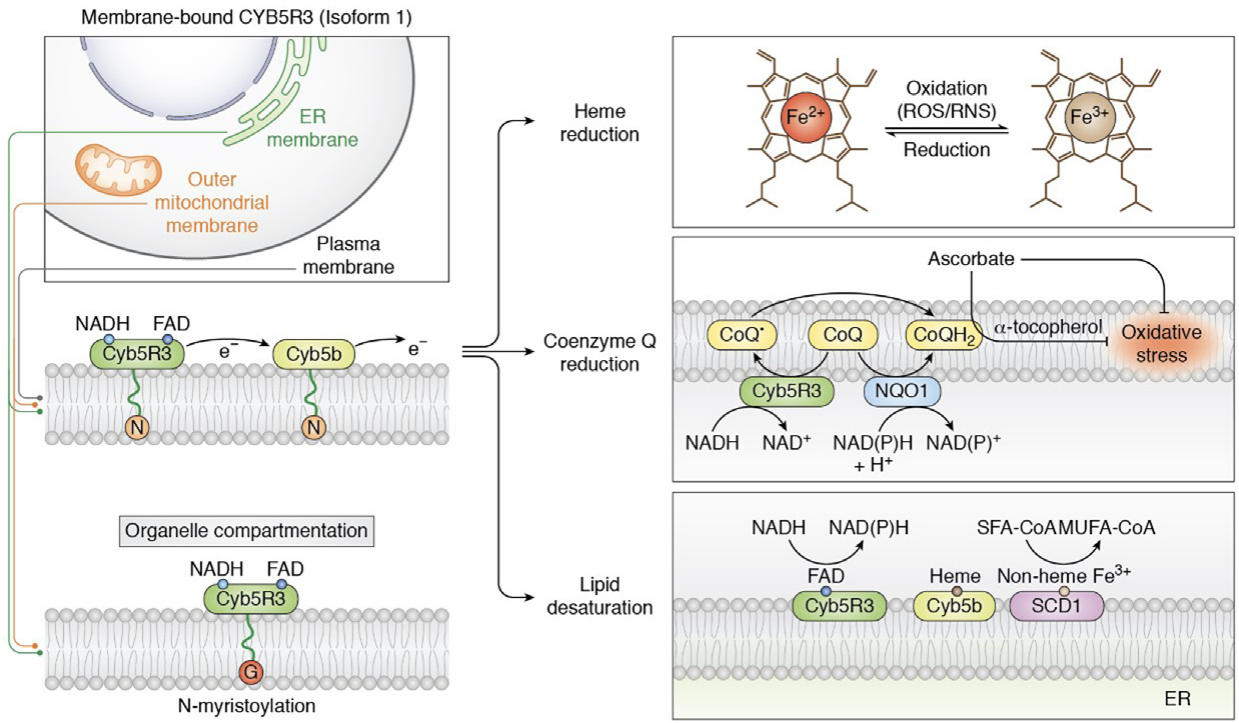
**Membrane-bound CYB5R3 is compartmentalized to the ER, OMM, and PM and catalyzes the transfer of electrons from NADH to target substrate.** CYB5R3 reduces heme iron and oxidized CoQ in biological membranes to mitigate oxidative stress. In the ER, CYB5R3 reduces stearoyl-CoA desaturase-1 (SCD1) through transferring electrons to the electron mediator cytochrome b_5_ (isoform B). Reduction of SCD1 results in the desaturation of saturated fatty acids (SFAs) to monounsaturated fatty acids (MUFAs). The N and G circles (*left*) reflect the N-myristoyl anchor and glycine residue by which N-myristoylation occurs, respectively.

CYB5R3 has been extensively studied, concluding that this enzyme is vital for preserving cellular redox equilibrium. CYB5R3 is implicated in several redox reactions that affect lipid metabolism, cholesterol biosynthesis, drug metabolism, oxidative stress, and heme reduction (Table 1) (11). There are two isoforms of CYB5R3; a soluble, 31 kDa isoform located in the cytosol of erythrocytes (36) and a membrane-bound, 34 kDa isoform tethered to the ER, plasma membrane, and outer mitochondrial membrane *via* a myristoyl group (Fig. 3) in somatic cells (37, 38, 39). The *N*-myristoylation of the membrane-bound isoform occurs on a glycine residue located at position 2 (38). The catalytic domains of CYB5R3 are indistinguishable in the soluble and membrane isoforms (40), differing only in the *N*-terminus (39), which is spliced in the soluble isoform. Both isoforms of CYB5R3 contain two domains: an NADH and an FAD-binding domain. The FAD domain, consisting of six antiparallel β-sheets and one α-helix, seats a large cleft where the FAD prosthetic group is situated. The NADH domain, comprised of five β-strands and four α-helices, harbors a pocket in which NADH associates (10). These two domains are connected by a linker region which embodies three antiparallel β-sheets (10). Upon NADH binding to CYB5R3, which occurs in less than 2 milliseconds (41), a conformational shift occurs in both the NADH and FAD domains that orients T66 closer to the N5 atom of FAD (42). T66 is an important amino acid that facilitates efficient electron transfer from NADH to FAD by stabilizing the FAD moeity (43). Moreover, molecular dynamics simulations have revealed that R91 has a favorable electrostatic interaction with bound FAD, while K110 is a crucial bridging residue between FAD and NADH, enabling reducing equivalents to be passed to target substrates (28). The domain rearrangement triggered by NADH binding creates a robust hydrogen-bonding network from the N5 of FAD to His49 and forms a stable stacking complex formed by the isoalloxazine ring of FAD and the nicotinamide ring of NAD^+^ (42). This stacking complex upholds the planarity of the isoalloxazine ring and permits efficient electron transfer to the noncovalently bound FAD molecule (42). Electrons transferred to FAD are subsequently passed to target substrates, where CYB5 is typically the first recipient of reducing equivalents (44).

### Prominent functions and regulation of CYB5R3

CYB5R3 is considered a “master regulator” of redox balance in cells by catalyzing numerous reduction reactions. Membrane-bound CYB5R3 controls several biological reduction reactions, including CoQ reduction (45, 46), heme reduction (11, 47, 48, 49), and lipid elongation and desaturation (50, 51) (Table 1). The most characterized is lipid metabolism in the liver, where CYB5R3 participates in fatty acid elongation and desaturation (Fig. 3) (8). ER-localized CYB5R3 ideally positions the enzyme where fatty acid desaturation and elongation transpires, permitting the CYB5R3-dependent reduction of stearoyl-CoA desaturase-1 (Fig. 3). Mouse studies have shown that overexpressed CYB5R3 increased long chain unsaturated fatty acids, commensurate with a longer lifespan (8). However, it is worth noting that no investigations have shown that endogenous CYB5R3 regulates fatty acid desaturation and elongation. Instead, these early studies only implicated CYB5R activity or peptide fragments with such an activity in lysates, usually the liver (52). The enzyme with CYB5R activity was later inferred to be on chromosome 22 where human *CYB5R3* is located based on 2,6-dichlorophenolindophenol reduction activity (53). Despite our vast knowledge on CYB5R3, many questions endure regarding its redox regulatory roles during divergent physiological or pathological conditions.

CYB5R3 is posttranslationally modified *via* phosphorylation, ubiquitination, and acetylation at several amino acid residues, the most abundant being phosphorylation at Y80 (21). Y80 is situated in the FAD domain near the FAD-binding site, implying that phosphorylation at this site might govern electron transfer efficacy to the FAD. It is possible that phosphorylation at Y80 mediates CYB5R3 activity by enhancing or reducing enzymatic activity. Yet, functional studies interrogating how Y80 phosphorylation impacts CYB5R3 activity and its relationship with disease have not been reported. Future studies dedicated to understanding PTMs at Y80 could provide valuable information into CYB5R3 regulation and its role in physiology and disease.

### CYB5 as an essential substrate

One of the most crucial protein partner substrates of CYB5R3 is CYB5. The CYB5 family plays a key role in mediating the electron transfer reaction carried out by CYB5R3 (Table 2). CYB5 is a small, ubiquitously expressed heme protein found in plants, animals, and fungi, functioning as an electron transporter in a plethora of reactions (54). Studies in plants, yeast, and mammals have demonstrated that CYB5 can also accept electrons from NADPH:cytochrome P450 reductase (13). In vertebrates, there are two isoforms of CYB5: CYB5A, anchored to the endoplasmic reticulum membrane, and CYB5B, anchored to the outer mitochondrial membrane (55). Both isoforms contain a heme-binding domain with near-identical folds, comprised of six alpha-helices and five beta-sheets (17, 18). CYB5 and CYB5R3 are ubiquitously expressed proteins generally involved in NADH-dependent electron transport, where CYB5R3 transfers two reducing equivalents from NADH to FAD situated in the FAD-binding domain, then ultimately to CYB5 (44). A study with purified enzyme showed that CYB5R3 binds to CYB5 with a K_m_ of 20 μM and reaches a V_max_ of 272 μmol min^−1^ mg^−1^ when NADH is used as an electron donor (30). One study revealed, through site-directed mutagenesis, that electrostatic interactions between lysine residues in CYB5R3 and the carboxyl groups in CYB5 maintains the two proteins in a tight complex for electron transfer (56). Critically, the reduction of FAD is the rate-limiting step in the electron transfer from CYB5R3 to CYB5 (43). CYB5 acts as an electron transfer mediator during CYB5R3-catalyzed reactions as shown through P450 monooxygenation (51), fatty acid desaturation and elongation (57, 58), myoglobin reduction (59), cytoglobin reduction (48, 59, 60), and hemoglobin reduction (61). However, CYB5R3 has been shown to function without CYB5, as shown with electron transfer to CoQ to stabilize ascorbate (45, 62, 63), the recycling of plasma membrane vitamin E (64), and the protection against ceramide-induced apoptosis (65).

**Table 2:** Known associated partners and substrates of human CYB5R3

Associated partners and substrates	Primary subcellular location(s)	Tissue location(s)	Functional role	References
Cytochrome b_5_ (isoforms A and B)	Mitochondria, cytosol	Ubiquitous	Electron acceptor and carrier	(54, 55)
Soluble guanylate cyclase	Cytosol	Primarily in heart tissue, particularly vascular smooth muscle	Electron acceptor leading to heme iron reduction, catalyzes reduction of GTP to cGMP	(4, 19, 49, 138)
Mitochondrial amidoxime reducing component (mARC)	Mitochondria	Ubiquitous	Electron transfer reaction, catalyzes the reduction of N-oxygenated molecules, drug metabolism	(89, 90, 91, 92)
Coenzyme Q	Mitochondria	Ubiquitous	Electron-transferring membrane protein complex in the mitochondrial respiratory chain, protection against lipid peroxidation	(73, 139)
Hemoglobin	Cytosol, extracellular	Ubiquitous, but most highly expressed in the blood	Electron acceptor leading to heme iron reduction, oxygen transport to peripheral tissues	(19, 87, 97)
Myoglobin	Cytosol, extracellular	Primarily cardiac and skeletal muscle	Storage and transport of oxygen from the cell membrane to the mitochondria, nitric oxide regulation	(19, 59, 85)
Mitoregulin	Mitochondria	Primarily cardiac and skeletal muscle, adipose tissue	Regulates mitochondrial complex assembly and respiration rate, controls mitochondrial ROS levels, maintains cellular lipid composition	(19, 95)
FoxO1	Mitochondria, nucleus, cytosol	Primarily skeletal muscle	Insulin signaling, regulation of metabolic homeostasis in response to oxidative stress	(19, 107, 108, 109, 140)
Molecular oxygen	Ubiquitous	Ubiquitous	Oxidative phosphorylation	(141, 142)
VDAC1	Mitochondria	Primarily skeletal muscle	Facilitates the transport of metabolites and ions across the outer mitochondrial membrane	(19, 79, 81, 82)
NOX4	Plasma Membrane, nucleus, mitochondria, ER	Primarily in the kidney, artery	Oxygen sensor, catalyzes the reduction of molecular oxygen to ROS	(3, 69, 143, 144)
Ascorbate	Ubiquitous	Ubiquitous	Potent antioxidant	(80, 145)
Cytoglobin	Cytosol	Ubiquitous	Facilitates oxygen transport, protection against oxidative stress, NO scavenging	(48, 59, 60, 61, 88)

The main subcellular and tissue location, as well as the functional role, of each substrate/partner are shown.

### The roles of CYB5R3 in mediating redox balance and oxidative stress

A multitude of studies have identified key substrates of CYB5R3 that play a role in mediating nitric oxide (NO) and ROS signaling. One example is soluble guanylate cyclase (sGC) in vascular smooth muscle cells (Table 2). sGC is activated upon binding of NO, catalyzing the formation of cyclic GMP, leading to blood vessel dilation. The major prerequisite for NO-induced sGC activation is reduced heme iron (Fe^2+^) in the sGC β H-NOX domain (49). In the presence of oxidants, sGC can become oxidized and insensitive to NO, a state that contributes to a myriad of diseases (66, 67, 68). sGC heme iron is maintained in its Fe^2+^ state *via* direct interaction with CYB5R3, thereby regulating cGMP signaling needed for downstream activation of protein kinase G–dependent signaling and blood vessel dilation (49). Studies with purified enzyme demonstrated that sGC heme iron is reduced by CYB5R3 with a rate constant of 1.56 × 10^4^ M^−1^ min^−1^ (29). Two studies demonstrated that mice with CYB5R3 deficiency in vascular smooth muscle cells exhibited increased mean arterial systemic pressure as a consequence of impaired sGC reduction in angiotensin II–induced hypertension and in sickle cell disease (4, 9). These studies demonstrated that targeting the CYB5R3-sGC axis could alleviate the poor outcomes associated with cardiovascular diseases. CYB5R3 has also been shown to cooperate with NADPH oxidase 4 (NOX4), an NADPH oxidase that reduces molecular oxygen to generate predominately hydrogen peroxide in vascular endothelial cells on the outer mitochondrial membrane (Table 2) (3). It has been demonstrated that CYB5R3 bolsters NOX4-derived hydrogen peroxide production at the outer mitochondrial membrane (69) and is optimal when coupled with CoQ (3). Importantly, CYB5R3’s regulation of NOX4-dependent hydrogen peroxide production reduces vascular wall inflammation and tempers inflammatory signaling. This newfound molecular interaction provides key insight into possible therapeutic options for clinical management of inflammatory diseases, potentially in patients who possess loss-of-function mutations in the *CYB5R3* gene (3).

It is widely accepted that CYB5R3 plays an important role in antioxidant stress responses and can be viewed as a “resilience enzyme” that protects cells from stress. Under stress conditions, CYB5R3 maintains membrane embedded α-tocopherol and ascorbate, potent membrane antioxidants in living cells, in their reduced state (Fig. 3 and Table 2) (70, 71, 72). This is achieved through the CYB5R3-catalyzed reduction of CoQ in biological membranes (Fig. 3). CoQ is a molecule present in all cells and membranes, where it functions not only as an important electron carrier in the mitochondrial respiratory chain (73) but also as a potent antioxidant that can independently quench ROS or through reducing α-tocopherol and ascorbate free radical (AFR) (Table 2) (74). As such, CYB5R3 reduction of CoQ is essential to the membrane antioxidant pathway (1, 19, 75) for protection against lipid peroxidation, a process in which oxidants attack polyunsaturated fatty acid phospholipids leading to the degradation and subsequent perturbation of cell membranes (76). Thus, maintaining CoQ and α-tocopherol in their reduced states is essential for antioxidant protection. The conversion of AFR to ascorbate, a potent antioxidant (77), is also pivotal in protecting cells against lipid peroxidation (78), a reaction carried out by CYB5R3 as previously described. Although not a direct interaction, CYB5R3 and voltage-dependent anion-selective channel 1 (VDAC1); an outer mitochondrial membrane protein responsible for the passage of metabolites, ions, and nucleotides into the mitochondria (79); have been shown to work in tandem as a “redox-cycling system” to mediate AFR transport (Table 2) (80). In addition to oxidative stress suppression, the reduction of AFR to ascorbate restores the cellular ascorbate pool and maintains the cellular NAD^+^/NADH ratio (80), both of which are critical for governing the intracellular redox state and metabolic processes. VDAC1 controls membrane integrity while also governing the flow of AFR into the mitochondrial matrix (81, 82). Given the indirect nature of CYB5R3-VDAC1 interactions, further studies must be performed to characterize alternative signaling mediators and pathways that could be involved in the “redox-cycling system” to mitigate excessive oxidative stress.

### Globins as primary substrates for CYB5R3

Among CYB5R3’s most important substrates in human physiology are globins, a family of heme-containing globular proteins necessary for oxygen transport to tissues (Table 2). The soluble form of CYB5R3 is responsible for the reduction of methemoglobin to hemoglobin in erythrocytes, permitting adequate oxygen binding and delivery to downstream tissues (83, 84). In this reaction, CYB5R3 reduces the ferric iron within methemoglobin to convert it to ferrous iron (12). The reduction of FAD is a rate-limiting step in this electron transfer reaction (12, 43). Importantly, the reduction of methemoglobin is not exclusive to the soluble isoform of CYB5R3. Straub *et al.* demonstrated a novel paradigm between membrane-bound CYB5R3 and alpha globin expressed in arterial endothelial cells. At the myoendothelial junction, the heme iron of alpha globin is redox-regulated by CYB5R3 to control NO diffusion and vascular tone (47). This study highlights that CYB5R3 plays a significant role in mediating globin redox state not only in erythrocytes but also in arterial endothelial cells. Additionally, CYB5R3 also directly reduces myoglobin, a globin hemoprotein that possesses a reactive heme iron for binding oxygen and subsequent transport from the plasma membrane to the mitochondria in muscle fibers (85). When the iron is oxidized to its ferric form (metmyoglobin), oxygen binding is mitigated and oxidative phosphorylation is hampered (86). CYB5R3 is responsible for reducing metmyoglobin iron to its ferrous state, which maintains the physiological role of myoglobin in muscle tissue (86). Lastly, cytoglobin can also be reduced by CYB5R3. Cytoglobin is similar to hemoglobin and myoglobin containing a hexacoordinate heme that facilitates oxygen transport and protects against oxidative stress (87). The reduction reaction involves CYB5B as an intermediate substrate of CYB5R3 in the transfer of electrons to cytoglobin (88). The existence of this reaction has been illustrated in vascular smooth muscle cells, with cytoglobin playing a key role in regulating blood pressure and vascular tone *via* NO-scavenging mechanisms (48, 60). Interestingly, the reduction of purified, human cytoglobin occurs at an order of magnitude faster than other heme-containing globins (48, 60, 61). Although this interaction between CYB5R3 and globins has been demonstrated indirectly, the direct interaction has not been shown experimentally. In fact, loss of CYB5R3 in smooth muscle cells *in vivo* did not impact sodium nitroprusside–stimulated vasodilation, suggesting that there is likely another reductase controlling cytoglobin-mediated NO scavenging in the compensation for the loss of CYB5R3 (4). Currently, it is unclear whether the reduction of all globins by CYB5R3 requires CYB5 as an electron intermediate. It is worth investigating whether hemoglobin and myoglobin, like cytoglobin, also require CYB5 as the intermediate electron carrier *in vitro* and *in vivo*. Additionally, it remains to be determined whether other reductases in the CYB5R family directly reduce hemoglobin or require CYB5 and how this may be different based on tissue and cell type. Future studies aimed at investigating these possibilities are warranted to better understand how the CYB5R family of enzymes might govern globin reduction.

### Mitochondrial-associated partners of CYB5R3

Membrane-bound CYB5R3 can localize to the outer mitochondrial membrane. As such, several mitochondrial-associated partners of CYB5R3 have been discovered, such as mitochondrial amidoxime reducing component (mARC) and mitoregulin (Mtln). Studies show that CYB5R3 interacts with mARC, a mammalian molybdenum-containing enzyme that exists in two isoforms, mARC1 and mARC2 (89), and catalyzes the reduction of *N*-oxygenated and *N*-hydroxylated structures, respectively (Table 2) (90). However, cell culture studies show that electron transfer by mARC requires its strong interaction with CYB5B alone or both CYB5B and CYB5R3 (91, 92). The synergy of mARC and CYB5R3, and in some instances also CYB5, is essential for regulating *N*-reductive drug metabolism in human cells (93). It is unclear whether the other CYB5R family members play a role in this *N*-reductive system. CYB5R3 also interacts with the 56 amino acid-long peptide Mtln (Table 2). Mtln is encoded by the gene *LINC00116* and is localized to the mitochondria. Mtln is also important for mitochondrial respiratory complex I activity (94), decreasing mitochondrial ROS (95), and forming mitochondrial super complexes (95). CYB5R3 interacts with Mtln at the mitochondrial membrane, where it likely maintains lipid homeostasis, metabolism, and integrity of the mitochondrial membrane, given its role in fatty acid desaturation and cholesterol biosynthesis (58, 94). It was speculated that Mtln acts to stabilize CYB5R3 and protects it from partial or complete proteolysis (94) but it has only been found to bolster CYB5R3 activity related to lipid metabolism through an unknown mechanism. It is worth mentioning that the authors do not distinguish between a direct or indirect interaction. The conclusion that CYB5R3 interacts with Mtln was based largely on copurification studies. To support their findings, additional cell-based and functional assays assessing interactions, mitochondrial efficiency, and membrane composition should be performed to ascertain whether the two enzymes interact at the mitochondrial membrane.

### CYB5R3’s role in cardiovascular disease

**Figure 4:**
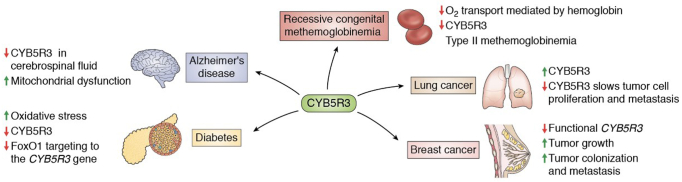
**The known implications of CYB5R3 in human health and disease**.

CYB5R3 deficiency is linked to cardiovascular disease. Among the most well-characterized diseases caused by CYB5R3 deficiency is recessive congenital methemoglobinemia (RCM), a hereditary disease where the oxygen carrying capacity of hemoglobin is compromised in erythrocytes (Fig. 4) (96). Nearly 60 different human genetic variants for *CYB5R3* have been reported with evidence of a substantial role in the pathogenesis of RCM (36) (Fig. 1). Loss-of-function mutations in *CYB5R3* increase erythrocytic methemoglobin levels, limiting oxygen binding and delivery to tissues (97). RCM exists in two forms: type 1 and type 2. Type 1 is caused primarily by missense mutations and that gives rise to an enzymatically active but unstable CYB5R3 protein. Type 1 manifests as cyanosis (36), an abnormal discoloration of the skin caused by high levels of deoxygenated ferric hemoglobin (98). Patients with type 1 RCM, however, present with normal life expectancy and no neurological symptoms (36). In contrast, type 2 RCM exhibits serious consequences. Caused by full stop or deletions that enzymatically inactivate CYB5R3, patients present with severe cyanosis and neurological deterioration, progressive microencephaly, and growth retardation (36). Interestingly, full stops or deletions are commonly located in the FAD-binding sites of CYB5R3 (99). An additional study conducted by Carew *et al.* (100) demonstrated that the cardiomyocyte-specific deletion of CYB5R3 in male mice causes cardiac hypertrophy and sudden cardiac death. These phenotypic differences are accompanied by elevated oxidative stress, decreased CoQ levels, and hemoprotein dysregulation in mouse CYB5R3-cardiomyocyte–specific knockout hearts (100). From a translational point of view, Carew *et al.* (100) revealed that a high-frequency missense genetic variant of CYB5R3, T117S, is associated with decreased event-free survival in those with African ancestry suffering from heart failure with reduced ejection fraction. It was shown that the membrane-bound T117S variant exhibits 50% reduced enzymatic activity when compared to WT CYB5R3. Together, this study demonstrates that CYB5R3 is critical for cardiomyocyte function and that the T117S CYB5R3 variant could be utilized as a genetic biomarker for persons of African ancestry that may be susceptible to an increased risk of death from heart failure with reduced ejection fraction.

### CYB5R3 and neurodegeneration

CYB5R3 has also been implicated in neurodegenerative disorders, such as Alzheimer’s disease (Fig. 4). Mitochondrial dysfunction is an established feature of Alzheimer’s disease (101, 102) and in the frequently utilized 5xFAD mouse model, a model that has a total of five Alzheimer’s disease–linked mutations: the Swedish (K670N/M671L), Florida (I716V), and London (V717I) mutations in APP and the M146L and L286V mutations in PSEN1 (103). 5xFAD mice exhibited reduced CYB5R3 levels in cerebrospinal fluid (104). The authors assumed that decreased CYB5R3 levels in the cerebrospinal fluid is commensurate with a decrease in mitochondrial number, but they did not consider that CYB5R3 also localizes to the ER and plasma membrane, in addition to the mitochondria. It is possible that Alzheimer’s disease etiology involves a causal effect for loss of CYB5R3 in mitochondrial dysfunction; however, more studies in the neural system must be performed to establish such a relationship.

### CYB5R3 and diabetes

Evidence has shown that CYB5R3 also plays a prominent role in pancreatic beta cell function (Fig. 4). Type II diabetes is associated with pancreatic beta cell failure, resulting in insulin resistance and inadequate glucose sensing. As such, pancreatic beta cells are unable to maintain insulin production, leading to a reduction in beta cell mass and function (105, 106). Several pathways have been identified in beta cell failure; of particular importance is the protective response orchestrated by the transcription factor FoxO1 (107, 108). Failure of FoxO1 to induce this stress response leads to mitochondrial dysfunction (109). It was discovered that CYB5R3, the main CYB5R isoform expressed in pancreatic beta cells in humans (110), is a target of FoxO1. It is possible that when FoxO1 targeting of *CYB5R3* is dysregulated, beta cell mitochondrial electron transport chain functions will be underprotected and mitochondrial ROS production excessive (Table 2) (5). This could lead to overt oxidative stress that is deleterious for pancreatic beta cell function.

### CYB5R3 and cancer

CYB5R3 has also been implicated in various cancers over the past decade (Fig. 4) (2, 111, 112). Several studies have highlighted CYB5R3 overexpression in cancer cells to protect against oxidative stress and apoptosis (2, 111, 112). Several research groups demonstrated that CYB5R3 overexpression and polymorphisms increase the risk of breast cancer in women, especially women of African ancestry (2, 112). The risk of *CYB5R3* polymorphism-associated breast cancer is further exacerbated in females who smoke cigarettes. Since CYB5R3 plays a role in drug metabolism (11), it is possible that the loss-of-function polymorphisms in *CYB5R3* result in accumulated carcinogen and cellular damage (2). Moreover, CYB5R3 overexpression is described in cancer cells of the lung. Genetic knockdown of CYB5R3 in lung cancer cells revealed slow proliferation and metastasis but did not affect cancer cell survival, pointing to a potential link between CYB5R3 and lung cancer (112). It is worth noting, however, that this study also reported a contradictory finding with CYB5R3 overexpression contributing to increased tumor size. Interestingly, the same study showed that CYB5R3 deficiency was deleterious in breast cancer, as evidenced by increased tumor colonization and metastasis. This could be due to differential cell lines utilized in these studies. These findings might also suggest that CYB5R3 has a differential role among tissues and, therefore, the progression of different types of cancers.

### CYB5R3 as a therapeutic target: Recent advances

Given the implications of CYB5R3 in human physiology and disease, efforts have been dedicated to devising CYB5R3-targeted therapeutics. One study demonstrated that treating HEK293 and rat renal endothelial cells with propylthiouracil derivatives ZINC05626394 (IC_50_ = 10.81 μM) and ZINC39395747 (IC_50_ = 9.14 μM) inhibit CYB5R3 activity by roughly 75%. In addition, acutely administered ZINC39395747 increased NO bioavailability in renal vascular cells, augmented renal blood flow, and reduced systemic blood pressure in hypertensive rats (11). However, it is not entirely clear whether these inhibitors block other NADH reductases *in vivo*, such as the other CYB5R family members. More selective CYB5R3 inhibitors could be a promising treatment for acutely modulating blood pressure (11).

As previously described, CYB5R3 plays an essential role in cellular redox and metabolic homeostasis, a hallmark of longevity (113, 114). As such, several pharmacological and genetic-based approaches have been developed to target CYB5R3 in hopes of extending lifespan and delaying age-related diseases associated with metabolic and redox imbalances, such as Alzheimer’s and Parkinson’s disease (8, 115, 116, 117). Overexpression of CYB5R3 in mouse models leads to extended lifespan, bolstered physical performance, ameliorated chronic inflammation, and protection against carcinogenesis (6). These findings are commensurate with CYB5R3’s role in generating intracellular NAD^+^ for utilization by sirtuins, NAD^+^-dependent histone deacetylases that are essential for DNA repair, controlling inflammation, and antioxidant defenses (6, 118, 119, 120). The NAD^+^/NADH ratio is regulated, in part, by CYB5R3 and is vital for cellular homeostasis, where too low of a ratio is associated with higher sensitivity to oxidative stress (121). Given CYB5R3’s role in age-related processes, drugs aimed at boosting CYB5R3 activity chronically and modulating the NAD^+^/NADH ratio in the cytosol and mitochondria (7) could be promising for treating age-related metabolic diseases (122, 123, 124) and maintaining cellular redox balance to prevent disease onset or severity.

## CYB5R4

Aside from CYB5R3, CYB5R4, also known as Ncb5or, is the most extensively studied in the CYB5R family. CYB5R4 is a 59 kDa flavohemoprotein that is ubiquitously expressed only in animal tissues and is the largest and most structurally unique in the CYB5R family, consisting of three distinct domains. CYB5R4 is the only CYB5R family member to contain a cytochrome b5 domain with coordinated heme that is lodged between two alpha-helices (55). The *N*-terminal b5 domain is linked to the C-terminal b5R domain *via* a CS (CHORD-SGT1) domain, comprised of roughly 90 amino acid residues and nine β-sheets (55). The CS domain of CYB5R4 differs from its structural homologs, featuring an additional β-sheet structure involving residues G256 and P267, forming two strands (β8 and β9) separated by a five-residue loop that orient in an antiparallel fashion (125). Beyond P267, a classic type I β-turn from R268 and T271 forms a linkage to the b5R domain (125). Interestingly, CS domains exist in diverse proteins and are commonly involved in protein–protein interactions, contributing to the potential diverse functions of CYB5R4 (126). Relative to CYB5R3, the B5R domain of CYB5R4 contains notable gaps and insertions, further illustrating the unique structural character of CYB5R4 (127). The cytochrome b5R domain binds FAD and NAD(P)H prosthetic groups, both serving as important enzymatic cofactors in the electron transfer reaction (128). The unique ability of CYB5R4 to utilize both NADH and NADPH is supported by a recent structural study on its FAD domain (125).

Unlike the other CYB5R family members, CYB5R4 is a soluble protein that localizes on the ER membrane (129). However, it should be noted that a more recent study failed to validate CYB5R4’s ER localization (130). The authors demonstrated that CYB5R4 is positioned in the cytosol and they speculated that CYB5R4 is not an integral protein anchored to the ER membrane but can be transiently recruited from the cytosol for fatty acid desaturation (130). The condition and mechanism for CYB5R4’s recruitment to the ER remains to be investigated (130). Given the multidomain structure of CYB5R4, full-length CYB5R4 has been resistant to crystallization; however, high-resolution crystal structures of the individual domains have been discovered (55). Future studies aimed at characterizing CYB5R4 structure are warranted to verify the absence of a membrane anchor domain. This would provide insight as to how CYB5R4 localization differs based on cell type and how CYB5R4 function changes as a consequence.

CYB5R4 is ubiquitinated and phosphorylated at numerous amino acid residues, the most abundant being phosphorylated at S471 and S476 (21). These two residues are situated in the NADH domain near the NADH-binding site, suggesting that the phosphorylation of these residues could impact either NADH binding or the efficiency of electron transfer from NADH to FAD. CYB5R4 has also been shown to be ubiquitinated at K277 and K442, two important modifications that could impact CYB5R4 stability and functional activity (21). One might speculate that under certain physiological conditions or stressors, ubiquitination at K277 and K442 could serve to target CYB5R4 to the proteasome for degradation. This could be due to either excess CYB5R4 that is unnecessary for the cell or mutated CYB5R4 that is deleterious for normal physiological processes. It is also possible that ubiquitination at these sites could alter protein localization or interacting partners. Because the function of these identified PTMs remains uncertain, future research dedicated to understanding PTMs of CYB5R4 is needed to understand the details in CYB5R4 regulation at a posttranslational level and its contribution to disease.

Kinetic measurements have demonstrated that human CYB5R4 can reduce numerous substrates *in vivo*, such as cytochrome c, methemoglobin, molecular oxygen, and ferricyanide (127, 129). CYB5R4 also reduces its own heme moiety through the consumption of NAD(P)H, where the FAD group bound at the reductase domain is necessary for mediating electron transfer from NAD(P)H to the heme moiety (127). Electrons are subsequently transferred to oxygen resulting in the generation of superoxide that can be dismutated to hydrogen peroxide.

Several studies have suggested that the loss of CYB5R4 results in diabetes mellitus as evidenced by mitochondrial dysfunction, disrupted ion channel signaling and iron homeostasis, and the progressive loss of white adipose tissue in the liver (130, 131). Notably, knockout of CYB5R4 caused early-onset diabetes in mice, irrespective of peripheral insulin sensitivity (132). CYB5R4 likely plays an important role in protecting pancreatic beta cells against oxidative stress by preventing the accumulation of ROS, similar to CYB5R3 (132). These unique findings identify a unique enzyme in CYB5R4 that could be targeted therapeutically for those suffering from diabetes mellitus. While the studies on CYB5R4 are certainly the most abundant in the CYB5R family, aside from CYB5R3, our mechanistic understanding of this enzyme is still incomplete. Therefore, interrogating the mechanisms involved in CYB5R4-mediated pancreatic beta cell protection must be performed to facilitate the development of high-quality therapies for diabetes mellitus. Moreover, one publication investigated the role of ER-associated CYB5R4 in mouse liver. They created a liver-specific CYB5R4 KO and found that free fatty acids, lipid catabolism, and oxidative stress are enriched in hepatocytes, characterized by increased mitochondrial content, PCG1 alpha expression, fatty acid oxidation rates, and oxidized glutathione content (133). In addition, CYB5R4 liver knockouts exhibited heightened lipotoxicity. These are novel findings and suggest that CYB5R4 may be a unique therapeutic target for those suffering with diabetes mellitus. Studies aimed at elucidating the mechanisms by which CYB5R4 might be mediating the pathogenesis of diabetes, and the assessment of potential interacting partners *in vitro* and *in vivo* would further facilitate the development of new therapeutic avenues. Finally, CYB5R4 deficiency has been characterized in the brain, where it was found that the conditional deletion of *CYB5R4* in the mouse cerebellum and midbrain results in altered iron homeostasis and locomotor activity and potentiates behavioral abnormalities (131, 134). Deletion of *CYB5R4* resulted in altered drinking and feeding behavior, neuroendocrine thirst regulation, and energy expenditure (131). Therefore, CYB5R4 could be playing a role in modulating the integrity of cerebellar regulation of satiety cues and voluntary exercise (131). The findings of this study combined with efforts to more rigorously understand the mechanisms by which CYB5R4 mediates cerebellar and midbrain processes could fuel pharmacological developments in this up-and-coming field of CYB5R4 research.

## CYB5R5

CYB5R5, also known as CYB5RL, is unequivocally the least studied in the CYB5R family. CYB5R5 is a 36 kDa flavoprotein that is the least expressed of the CYB5R family members (Table 1). While the predicted structure of CYB5R5 (Fig. 2) does not share the same structural motifs conserved across CYB5R1, CYB5R2, and CYB5R3, it is purported that CYB5R5 also utilizes both NADH and FAD as cofactors (Table 1). It is also uncertain whether CYB5R5 harbors a membrane anchor (Fig. 2). CYB5R5 is the least similar to CYB5R3, sharing a 27.87% sequence identity, and is the most similar to CYB5R4, sharing a 30.25% sequence identity. CYB5R5 has a very low tissue specificity and is therefore not enriched in any human tissue. CYB5R5 is predicted to be localized to the nucleoplasm and the ER but has not been proven experimentally. CYB5R5 is ubiquitinated and phosphorylated at K55 and T76, respectively (21, 135). These two residues are situated within the purported membrane anchor region near the beginning of the FAD domain. One might postulate that phosphorylation at T76 could potentially disrupt CYB5R5 membrane targeting and localization, which could inevitably interfere with normal CYB5R5 function. It is conceivable that ubiquitination at K55 could impact protein localization and protein–protein interactions and could also serve as a regulatory mechanism to stimulate the degradation of CYB5R5 in cases where CYB5R5 is not needed for a particular function or when CYB5R5 is nonfunctional. Future studies aimed to understand these PTM’s of CYB5R5 could not only help discern the crosstalk between these two sites but also provide crucial details as to how CYB5R5 activity is regulated at the protein level.

A study from Wang *et al.* uncovered a novel germline truncation mutation at R51 in CYB5R5, R51X, enriched in colon polyps in canines. With this mutation, CYB5R5 function is compromised, accelerating the generation of ROS and oxidative stress in the colon (136). In response, *Bacteroides uniformis*, an anaerobic bacterium that resides in the colon, expresses thioredoxin and nitroreductase, which together act as a bacterial redox system to mitigate oxidative stress induced by the R51X CYB5R5 mutation and the more aerobic environment of the jejunum (136). In turn, cell death is ameliorated, leading to the uncontrolled proliferation of cancer cells in the colon and the onset of extreme polyposis. Despite these findings, it is critical that future functional studies are conducted to validate these findings in canines and possibly extend similar studies to humans. Nevertheless, this study presents a significant first step into understanding the role of CYB5R5 in human health and disease, presenting a potential therapeutic target for colon cancer.

## Conclusions and future directions

A multitude of unanswered questions remain surrounding the biological roles of the CYB5R family of proteins. These include the following: (1) do the different CYB5R isoforms cooperate with one another or are their biological roles functionally separate and how do their roles differ based on tissue or cell type, (2) how are CYB5R proteins regulated at the transcriptional and posttranslational level and how does this govern enzymatic function, (3) are there genetic variants of the CYB5R isoforms that can be identified to predict high risk patients for different diseases, and (4) how can we leverage this information to design targeted CYB5R therapeutics? Collectively, these preexisting CYB5R studies have led to valuable insight emphasizing the importance of CYB5R enzymes in human physiology and disease. As further studies begin to uncover novel roles of CYB5R enzymes, emphasis should be placed on developing innovative strategies to therapeutically target CYB5R proteins to treat a plethora of diseases.

## Conflict of interest

Dr Straub is a consultant and stockholder for Creegh Pharmaceuticals. Dr Straub received research funds from Bayer Pharmaceuticals. All other authors declare that they have no conflicts of interest with the contents of this article.
